# *FANCM* missense variants and breast cancer risk: a case-control association study of 75,156 European women

**DOI:** 10.1038/s41431-022-01257-w

**Published:** 2023-01-27

**Authors:** Gisella Figlioli, Amandine Billaud, Thomas U. Ahearn, Natalia N. Antonenkova, Heiko Becher, Matthias W. Beckmann, Sabine Behrens, Javier Benitez, Marina Bermisheva, Marinus J. Blok, Natalia V. Bogdanova, Bernardo Bonanni, Barbara Burwinkel, Nicola J. Camp, Archie Campbell, Jose E. Castelao, Melissa H. Cessna, Stephen J. Chanock, NBCS Collaborators, Kristine K. Sahlberg, Anne-Lise Børresen-Dale, Inger Torhild Gram, Karina Standahl Olsen, Olav Engebråten, Bjørn Naume, Jürgen Geisler, Grethe I. Grenaker Alnæs, Kamila Czene, Peter Devilee, Thilo Dörk, Christoph Engel, Mikael Eriksson, Peter A. Fasching, Jonine D. Figueroa, Marike Gabrielson, Manuela Gago-Dominguez, Montserrat García-Closas, Anna González-Neira, Felix Grassmann, Pascal Guénel, Melanie Gündert, Andreas Hadjisavvas, Eric Hahnen, Per Hall, Ute Hamann, Patricia A. Harrington, Wei He, Peter Hillemanns, Antoinette Hollestelle, Maartje J. Hooning, Reiner Hoppe, Anthony Howell, Keith Humphreys, KConFab Investigators, David Amor, Lesley Andrews, Yoland Antill, Rosemary Balleine, Jonathan Beesley, Ian Bennett, Michael Bogwitz, Leon Botes, Meagan Brennan, Melissa Brown, Michael Buckley, Jo Burke, Phyllis Butow, Liz Caldon, Ian Campbell, Michelle Cao, Anannya Chakrabarti, Deepa Chauhan, Manisha Chauhan, Alice Christian, Paul Cohen, Alison Colley, Ashley Crook, James Cui, Eliza Courtney, Margaret Cummings, Sarah-Jane Dawson, Anna deFazio, Martin Delatycki, Rebecca Dickson, Joanne Dixon, Ted Edkins, Stacey Edwards, Gelareh Farshid, Andrew Fellows, Georgina Fenton, Michael Field, James Flanagan, Peter Fong, Laura Forrest, Stephen Fox, Juliet French, Michael Friedlander, Clara Gaff, Mike Gattas, Peter George, Sian Greening, Marion Harris, Stewart Hart, Nick Hayward, John Hopper, Cass Hoskins, Clare Hunt, Paul James, Mark Jenkins, Alexa Kidd, Judy Kirk, Jessica Koehler, James Kollias, Sunil Lakhani, Mitchell Lawrence, Jason Lee, Shuai Li, Geoff Lindeman, Lara Lipton, Liz Lobb, Sherene Loi, Graham Mann, Deborah Marsh, Sue Anne McLachlan, Bettina Meiser, Roger Milne, Sophie Nightingale, Shona O’Connell, Sarah O’Sullivan, David Gallego Ortega, Nick Pachter, Jia-Min Pang, Gargi Pathak, Briony Patterson, Amy Pearn, Kelly Phillips, Ellen Pieper, Susan Ramus, Edwina Rickard, Bridget Robinson, Mona Saleh, Anita Skandarajah, Elizabeth Salisbury, Christobel Saunders, Jodi Saunus, Rodney Scott, Clare Scott, Adrienne Sexton, Andrew Shelling, Peter Simpson, Melissa Southey, Amanda Spurdle, Jessica Taylor, Renea Taylor, Heather Thorne, Alison Trainer, Kathy Tucker, Jane Visvader, Logan Walker, Rachael Williams, Ingrid Winship, Mary Ann Young, Milita Zaheed, Agnes Jager, Anna Jakubowska, Elza K. Khusnutdinova, Yon-Dschun Ko, Vessela N. Kristensen, Annika Lindblom, Jolanta Lissowska, Jan Lubiński, Arto Mannermaa, Siranoush Manoukian, Sara Margolin, Dimitrios Mavroudis, William G. Newman, Nadia Obi, Mihalis I. Panayiotidis, Muhammad U. Rashid, Valerie Rhenius, Matti A. Rookus, Emmanouil Saloustros, Elinor J. Sawyer, Rita K. Schmutzler, Mitul Shah, Reijo Sironen, Melissa C. Southey, Maija Suvanto, Rob A. E. M. Tollenaar, Ian Tomlinson, Thérèse Truong, Lizet E. van der Kolk, Elke M. van Veen, Barbara Wappenschmidt, Xiaohong R. Yang, Manjeet K. Bolla, Joe Dennis, Alison M. Dunning, Douglas F. Easton, Michael Lush, Kyriaki Michailidou, Paul D. P. Pharoah, Qin Wang, Muriel A. Adank, Marjanka K. Schmidt, Irene L. Andrulis, Jenny Chang-Claude, Heli Nevanlinna, Georgia Chenevix-Trench, D. Gareth Evans, Roger L. Milne, Paolo Radice, Paolo Peterlongo

**Affiliations:** 1 IFOM ETS - The AIRC Institute of Molecular Oncology, Genome Diagnostics Program, Milan, Italy; 2 Division of Cancer Epidemiology and Genetics, National Cancer Institute, National Institutes of Health, Department of Health and Human Services, Bethesda, MD USA; 3 N.N. Alexandrov Research Institute of Oncology and Medical Radiology, Minsk, Belarus; 4 University Medical Center Hamburg-Eppendorf, Institute of Medical Biometry and Epidemiology, Hamburg, Germany; 5 University Hospital Erlangen, Department of Gynecology and Obstetrics, Comprehensive Cancer Center Erlangen-EMN, Friedrich-Alexander University Erlangen-Nuremberg, Erlangen, Germany; 6 German Cancer Research Center (DKFZ), Division of Cancer Epidemiology, Heidelberg, Germany; 7 Spanish National Cancer Research Centre (CNIO), Human Genetics Group, Madrid, Spain; 8 Centre for Biomedical Network Research on Rare Diseases (CIBERER), Instituto de Salud Carlos III, Madrid, Spain; 9 Institute of Biochemistry and Genetics of the Ufa Federal Research Centre of the Russian Academy of Sciences, Ufa, Russia; 10 Maastricht University Medical Center, Department of Clinical Genetics, Maastricht, the Netherlands; 11 Hannover Medical School, Department of Radiation Oncology, Hannover, Germany; 12 Hannover Medical School, Gynaecology Research Unit, Hannover, Germany; 13 IEO, European Institute of Oncology IRCCS, Division of Cancer Prevention and Genetics, Milan, Italy; 14 German Cancer Research Center (DKFZ), Molecular Epidemiology Group, C080 Heidelberg, Germany; 15 University of Heidelberg, Molecular Biology of Breast Cancer, University Womens Clinic Heidelberg, Heidelberg, Germany; 16 University of Utah, Department of Internal Medicine and Huntsman Cancer Institute, Salt Lake City, UT USA; 17 University of Edinburgh, Centre for Genomic and Experimental Medicine, Institute of Genetics & Cancer, Edinburgh, UK; 18 The University of Edinburgh, Usher Institute of Population Health Sciences and Informatics, Edinburgh, UK; 19 Instituto de Investigación Sanitaria Galicia Sur (IISGS), Xerencia de Xestion Integrada de Vigo-SERGAS, Oncology and Genetics Unit, Vigo, Spain; 20 Intermountain Healthcare, Salt Lake City, UT USA; 21 Karolinska Institutet, Department of Medical Epidemiology and Biostatistics, Stockholm, Sweden; 22 Leiden University Medical Center, Department of Pathology, Leiden, the Netherlands; 23 Leiden University Medical Center, Department of Human Genetics, Leiden, the Netherlands; 24 University of Leipzig, Institute for Medical Informatics, Statistics and Epidemiology, Leipzig, Germany; 25 University of Leipzig, LIFE - Leipzig Research Centre for Civilization Diseases, Leipzig, Germany; 26 The University of Edinburgh, Cancer Research UK Edinburgh Centre, Edinburgh, UK; 27 Fundación Pública Galega de Medicina Xenómica, Instituto de Investigación Sanitaria de Santiago de Compostela (IDIS), Complejo Hospitalario Universitario de Santiago, SERGAS, Genomic Medicine Group, International Cancer Genetics and Epidemiology Group, Santiago de Compostela, Spain; 28 University of California San Diego, Moores Cancer Center, La Jolla, CA USA; 29 Spanish National Cancer Research Centre (CNIO), Human Cancer Genetics Programme, Madrid, Spain; 30 Health and Medical University, Potsdam, Germany; 31 INSERM, University Paris-Saclay, Center for Research in Epidemiology and Population Health (CESP), Team Exposome and Heredity, Villejuif, France; 32 Helmholtz Zentrum München, German Research Center for Environmental Health, Institute of Diabetes Research, Neuherberg, Germany; 33 The Cyprus Institute of Neurology & Genetics, Department of Cancer Genetics, Therapeutics and Ultrastructural Pathology, Nicosia, Cyprus; 34 Faculty of Medicine and University Hospital Cologne, University of Cologne, Center for Familial Breast and Ovarian Cancer, Cologne, Germany; 35 Faculty of Medicine and University Hospital Cologne, University of Cologne, Center for Integrated Oncology (CIO), Cologne, Germany; 36 Södersjukhuset, Department of Oncology, Stockholm, Sweden; 37 German Cancer Research Center (DKFZ), Molecular Genetics of Breast Cancer, Heidelberg, Germany; 38 University of Cambridge, Centre for Cancer Genetic Epidemiology, Department of Oncology, Cambridge, UK; 39 Erasmus MC Cancer Institute, Department of Medical Oncology, Rotterdam, the Netherlands; 40 Dr. Margarete Fischer-Bosch-Institute of Clinical Pharmacology, Stuttgart, Germany; 41 University of Tübingen, Tübingen, Germany; 42 University of Manchester, Division of Cancer Sciences, Manchester, UK; 43 Pomeranian Medical University, Department of Genetics and Pathology, International Hereditary Cancer Center, Szczecin, Poland; 44 Pomeranian Medical University, Independent Laboratory of Molecular Biology and Genetic Diagnostics, Szczecin, Poland; 45 Bashkir State University, Department of Genetics and Fundamental Medicine, Ufa, Russia; 46 Department of Internal Medicine, Johanniter GmbH Bonn, Johanniter Krankenhaus, Bonn, Germany; 47 Department of Medical Genetics, Oslo University Hospital and University of Oslo, Oslo, Norway; 48 Karolinska Institutet, Department of Molecular Medicine and Surgery, Stockholm, Sweden; 49 Karolinska University Hospital, Department of Clinical Genetics, Stockholm, Sweden; 50 M. Sklodowska-Curie National Research Oncology Institute, Department of Cancer Epidemiology and Prevention, Warsaw, Poland; 51 University of Eastern Finland, Translational Cancer Research Area, Kuopio, Finland; 52 University of Eastern Finland, Institute of Clinical Medicine, Pathology and Forensic Medicine, Kuopio, Finland; 53 Kuopio University Hospital, Biobank of Eastern Finland, Kuopio, Finland; 54 Fondazione IRCCS Istituto Nazionale dei Tumori di Milano, Unit of Medical Genetics, Department of Medical Oncology and Hematology, Milan, Italy; 55 Karolinska Institutet, Department of Clinical Science and Education, Södersjukhuset, Stockholm, Sweden; 56 University Hospital of Heraklion, Department of Medical Oncology, Heraklion, Greece; 57 University of Manchester, Manchester Academic Health Science Centre, Division of Evolution and Genomic Sciences, School of Biological Sciences, Faculty of Biology, Medicine and Health, Manchester, UK; 58 St Mary’s Hospital, Manchester University NHS Foundation Trust, Manchester Academic Health Science Centre, North West Genomics Laboratory Hub, Manchester Centre for Genomic Medicine, Manchester, UK; 59 Shaukat Khanum Memorial Cancer Hospital and Research Centre (SKMCH & RC), Department of Basic Sciences, Lahore, Pakistan; 60 The Netherlands Cancer Institute - Antoni van Leeuwenhoek hospital, Division of Psychosocial Research and Epidemiology, Amsterdam, the Netherlands; 61 University Hospital of Larissa, Department of Oncology, Larissa, Greece; 62 King’s College London, School of Cancer & Pharmaceutical Sciences, Comprehensive Cancer Centre, Guy’s Campus, London, UK; 63 Faculty of Medicine and University Hospital Cologne, University of Cologne, Center for Molecular Medicine Cologne (CMMC), Cologne, Germany; 64 Kuopio University Hospital, Imaging Center, Department of Clinical Pathology, Kuopio, Finland; 65 Monash University, Precision Medicine, School of Clinical Sciences at Monash Health, Clayton, Victoria, Australia; 66 The University of Melbourne, Department of Clinical Pathology, Melbourne, Victoria, Australia; 67 Cancer Council Victoria, Cancer Epidemiology Division, Melbourne, Victoria, Australia; 68 University of Helsinki, Department of Obstetrics and Gynecology, Helsinki University Hospital, Helsinki, Finland; 69 Leiden University Medical Center, Department of Surgery, Leiden, the Netherlands; 70 The University of Edinburgh, Cancer Research Centre, Edinburgh, UK; 71 The Netherlands Cancer Institute - Antoni van Leeuwenhoek hospital, Family Cancer Clinic, Amsterdam, the Netherlands; 72 University of Cambridge, Centre for Cancer Genetic Epidemiology, Department of Public Health and Primary Care, Cambridge, UK; 73 The Cyprus Institute of Neurology & Genetics, Biostatistics Unit, Nicosia, Cyprus; 74 The Netherlands Cancer Institute, Division of Molecular Pathology, Amsterdam, the Netherlands; 75 Lunenfeld-Tanenbaum Research Institute of Mount Sinai Hospital, Fred A. Litwin Center for Cancer Genetics, Toronto, Ontario Canada; 76 University of Toronto, Department of Molecular Genetics, Toronto, Ontario, Canada; 77 University Medical Center Hamburg-Eppendorf, Cancer Epidemiology Group, University Cancer Center Hamburg (UCCH), Hamburg, Germany; 78 QIMR Berghofer Medical Research Institute, Department of Genetics and Computational Biology, Brisbane, Queensland Australia; 79 The University of Melbourne, Centre for Epidemiology and Biostatistics, Melbourne School of Population and Global Health, Melbourne, Victoria, Australia; 80 Fondazione IRCCS Istituto Nazionale dei Tumori (INT), Unit of Molecular Bases of Genetic Risk and Genetic Testing, Department of Experimental Oncology, Milan, Italy; 81 Department of Research, Vestre Viken Hospital, Drammen, Norway; 82 Department of Tumor Biology, Institute for Cancer Research, Oslo University Hospital - Radiumhospitalet, Oslo, Norway; 83 Department of Cancer Genetics, Institute for Cancer Research, Oslo University Hospital-Radiumhospitalet, Oslo, Norway; 84 Institute of Clinical Medicine, Faculty of Medicine, University of Oslo, Oslo, Norway; 85 Department of Community Medicine, The Arctic University of Norway, Tromsø, Norway; 86 Department of Oncology, Division of Surgery and Cancer and Transplantation Medicine, Oslo University Hospital-Radiumhospitalet, Oslo, Norway; 87 Department of Oncology, Akershus University Hospital, Lørenskog, Norway; 88 Norwegian University of Science and Technology, Trondheim, Norway; 89 Oslo University Hospital, Oslo, Norway; 90 Østfold Hospital, Sarpsborg, Norway; 91 Cancer Registry of Norway, Oslo, Norway; 92 University of Oslo, Oslo, Norway; 93 Vestre Viken Hospital Trust, Drammen, Norway; 94 Medical Geneticist, Genetic Health Services, Victoria Royal Children’s Hospital, Melbourne, VIC 3050 Australia; 95 Hereditary Cancer Clinic, Prince of Wales Hospital, Randwick, NSW 2031 Australia; 96 Department Haem and Medical Oncology, Peter MacCallum Cancer Centre, St Andrews Place East Melbourne, Melbourne, VIC 3002 Australia; 97 Department of Translational Oncology, C/- Department of Medical Oncology, Westmead Hospital, Westmead, NSW 2145 Australia; 98 Research Officer, Queensland Institute of Medical Research, Herston Road, Herston, QLD 4002 Australia; 99 Silverton Place, 101 Wickham, Terrace Brisbane, QLD 4000 Australia; 100 Familial Cancer Centre, The Royal Melbourne Hospital, Grattan Street Parkville, Melbourne, VIC 3050 Australia; 101 Clinical Nurse Specialist, Hereditary Cancer Centre, Prince of Wales Hospital, Barker St, Randwick, NSW 203 Australia; 102 NSW Breast Cancer Institute, PO Box 143, Westmead, NSW 2145 Australia; 103 Department of Biochemistry, University of Queensland, St. Lucia, QLD 4072 Australia; 104 Molecular and Cytogenetics Unit, Prince of Wales Hospital, Randwick, NSW 2031 Australia; 105 ICON Cancer Care, 2 Melville St, Hobart, TAS 7000 Australia; 106 Medical Psychology Unit, Royal Prince Alfred Hospital, Camperdown, NSW 2204 Australia; 107 Replication and Genome Stability, Cancer Division, Garvan Institute of Medical Research, 370 Victoria Street, Darlinghurst, NSW 2010 Australia; 108 Peter MacCallum Cancer Centre, St Andrew’s Place, East Melbourne, VIC 3002 Australia; 109 Tasmanian Clinical Genetics Service, Tasmanian Health Service Royal, Hobart Hospital GPO Box 1061, Hobart, TAS 7001 Australia; 110 Specialist Breast Cancer Surgery, Richmond, VIC 3121 Australia; 111 School of Psychology, Brennan McCallum (Building A18), University of Sydney, Sydney, NSW 2006 Australia; 112 St Vincents Hospital Cancer Genetics Clinic, The Kinghorn Cancer Centre, Sydney, NSW Australia; 113 Genetics Department, Central Region Genetics Service, Wellington Hospital, New Wellington, New Zealand; 114 Director of Gynaecological Cancer Research, St John of God Subiaco Hospital, 12 Salvado Road, Subiaco, WA 6008 Australia; 115 Department of Clinical Genetics, Liverpool Health Service, PO Box 103, Liverpool, NSW 2170 Australia; 116 Department of Clinical Genetics, Level 3E, Royal North Shore Hospital, St Leonards, NSW 2065 Australia; 117 Epidemiology and Preventive Medicine, Monash University, Prahan, VIC 3004 Australia; 118 Department of Pathology, University of Queensland Medical School, Herston, NSW 4006 Australia; 119 Molecular Genetics Unit, Peter MacCallum Cancer Centre, Melbourne, VIC Australia; 120 Department of Gynaecological Oncology, Westmead Institute for Cancer Research, Westmead Hospital, Westmead, NSW 2145 Australia; 121 Director, Clinical Genetics Austin Health, Heidelberg Repatriation Hospital, PO Box 5444, Heidelberg West, VIC 3081 Australia; 122 Associate Genetic Counsellor, Level 2, Block 51 Royal North Shore Hospital, North Shore, NSW 2408 Australia; 123 Central Regional Genetic Services, Wellington Hospital Private bag 7902, Wellington, New Zealand; 124 Clinical Chemistry, Princess Margret Hospital for Children, Box D184, Perth, WA 6001 Australia; 125 Department of Biochemistry and Molecular Biology, University of Queensland, St Lucia Qld, 4072 Australia; 126 Tissue Pathology, IMVS, Adelaide, SA 5000 Australia; 127 Molecular Diagnostic Development, Pathology Department, Peter MacCallum Cancer Centre, Melbourne East Melbourne, VIC 3002 Australia; 128 South West Family Cancer Clinic, Liverpool Hospital, Liverpool, NSW 1871 Australia; 129 Clinical Geneticist, Royal North Shore Hospital, Level 2, Vindin House, St Leonards, NSW 2065 Australia; 130 Epigenetics Unit, Department of Surgery and Oncology, Imperial College London, London, W12 0NN England; 131 Medical Oncology Department, Regional Cancer and Blood, Services, Level 1 Building 7, Auckland City Hospital, 2 Park Rd. Grafton, Auckland, 1023 New Zealand; 132 Psychosocial Cancer Genetics Research Group, Parkville Familial Cancer Centre, 305 Grattan Street, Melbourne, VIC 3000 Australia; 133 Pathology Department, Level 1, Peter MacCallum Cancer Centre, St Andrew’s Place, East Melbourne, VIC 3002 Australia; 134 School of Molecular and Microbial Sciences, University of Queensland, St Lucia, Qld 4072 Australia; 135 Professor of Medicine, Department of Medical Oncology, Prince of Wales Hospital, Randwick, NSW 2031 Australia; 136 Victorian Clinical Genetics Service, Royal Melbourne Hospital, Parkville, VIC 3052 Australia; 137 Queensland Clinical Genetic Service, Royal Children’s Hospital, Bramston Terrace, Herston, QLD 4020 Australia; 138 Clinical Biochemistry Unit, Canterbury Health Labs, PO Box 151, Christchurch, New Zealand; 139 Illawarra Cancer Centre, Wollongong Hospital, Private Mail Bag 8808, South Coast Mail Centre, Wollongong, NSW 2521 Australia; 140 Familial Cancer Clinic, Peter MacCallum Cancer Centre, St Andrews Place East Melbourne, Melbourne, VIC 3002 Australia; 141 Breast and Ovarian Cancer Genetics, Monash Medical Centre, 871 Centre Road, Bentleigh East, Clayton, VIC 3165 Australia; 142 Queensland Institute for Medical Research, Royal Brisbane Hospital, Post Office, Herston, QLD 4029 Australia; 143 Centre for M.E.G.A. Epidemiology, University of Melbourne, Level 1, 723 Swanston Street, Carlton, VIC 3010 Australia; 144 Parkville Familial Cancer Centre, Peter MacCallum Cancer Centre & The Royal Melbourne Hospital, Melbourne, VIC 3000 Australia; 145 Southern Health Familial Cancer Centre, Monash Medical Centre, Special Medicine Building, 246 Clayton Rd Clayton Victoria 3168, Clayton, Australia; 146 Clinical Geneticist, Genetic Health Services, Monash Medical Centre, Clayton, VIC 3168 Australia; 147 Clinical Genetics Departments, Central Regional Genetics Service, Wellington Hospital, Newtown, New Zealand; 148 Familial Cancer Service, Department of Medicine, Westmead Hospital, Westmead, NSW 2145 Australia; 149 Breast Endocrine and Surgical Unit, Royal Adelaide Hospital, North Terrace, Adelaide, SA 5000 Australia; 150 UQ Centre for Clinical Research, Level 6 Building 71/918, University of Queensland, The Royal Brisbane Women’s Hospital, Herston, QLD 4029 Australia; 151 Prostate Cancer Research Program, 19 Innovation Walk, Level 3, Monash University, Clayton, VIC 3800 Australia; 152 Epigenetics and Disease Laboratory, QIMR Berghofer Medical Research Institute, Brisbane, QLD 4006 Australia; 153 Centre for Epidemiology and Biostatistics, Melbourne School of Population and Global Health, The University of Melbourne Level 3, 207 Bouverie Street, Carlton, VIC 3053 Australia; 154 Breast Cancer Laboratory, Walter and Eliza Hall Institute, PO Royal Melbourne Hospital, Parkville, VIC 3050 Australia; 155 Medical Oncology and Clinical Haematology Unit, Western Hospital, Footscray, SA Australia; 156 Medical Psychology Research Unit, Room 332, Brennan MacCallum Building (A18), The University of Sydney, Camperdown, NSW 2006 Australia; 157 Head of the Translational Breast Cancer, Genomics and Therapeutics Laboratory, Medical Oncologists, The Peter MacCallum Cancer Centre, Melbourne, VIC 3000 Australia; 158 Westmead Institute for Cancer Research, Westmead Millennium Institute, Westmead, NSW 2145 Australia; 159 Kolling Institute of Medical Research, Royal North Shore Hospital, St Leonards, NSW 2065 Australia; 160 Department of Oncology, St Vincent’s Hospital, 41 Victoria Parade, Fitzroy, VIC 3065 Australia; 161 Western Health and Peter MacCallum Cancer Centre, Consultant, General, Breast and Melanoma Surgeon, St Andrew’s Place, East Melbourne, VIC 3002 Australia; 162 Genetic Services of Western, Level 3 Agnes Walsh House, 374 Bagot Road, Subiaco, WA 6008 Australia; 163 Tumour Development Group, Garvan Institute of Medical Research, The Kinghorn Cancer Centre, 370 Victoria St, Darlinghurst, NSW 2010 Australia; 164 Familial Cancer and Clinical Genetics, Royal Melbourne Hospital, Grattan Street, Parkville, VIC 3050 Australia; 165 Molecular Pathology Department, Peter MacCallum Cancer Centre, Melbourne, VIC 3000 Australia; 166 Clinical Geneticist, Genetic Services of Western Australia Women and Newborn Health Service Agnes Walsh House, King Edward, Memorial Hospital 374 Bagot Road, Subiaco, WA 6008 Australia; 167 Tas Clinical Genetics Service, Royal Hobart Hospital, GPO Box 1061, Hobart Tasmania, TAS 7001 Australia; 168 The Gene Council, Perth, Australia, PO Box 510, North Perth, WA 6906 Australia; 169 Department of Medical Oncology, Peter MacCallum Cancer Centre, St Andrew’s Place, East Melbourne, VIC 3002 Australia; 170 Associate Genetic Counsellor, Parkville Familial Cancer Centre and Genomic Medicine, VCCC Grattan Street, Melbourne, VIC 3000 Australia; 171 School of Women’s and Children’s Health Adult Cancer Program Level 2, Lowy Cancer Research Centre Cnr, High and Botany St, UNSW, Sydney, NSW Australia; 172 Familial Cancer Centre, Westmead Hospital, Westmead, NSW 2145 Australia; 173 Oncology Service, Christchurch Hospital Private Bag 4710, Christchurch, New Zealand; 174 Centre for Genetic Education, Prince of Wales Hospital, Randwick, NSW 2031 Australia; 175 Deputy Director General Surgery, The Royal Melbourne Hospital, Consultant Breast and Endocrine Surgeon, The Royal Melbourne Hospital and Peter Mac Callum Centre, The University of Melbourne, Melbourne, VIC Australia; 176 Anatomical Pathology, Conjoint Associate Professor, UNSW, Prince of Wales Hospital Randwick, Randwick, 2031 NSW Australia; 177 Director Breast Surgery, The Royal Melbourne Hospital, The Royal Melbourne Hospital and Peter Mac Callum Centre, The University of Melbourne, Melbourne, VIC Australia; 178 Breast Pathology, University of Queensland Centre for Clinical Research, Building 71/918 Royal Brisbane and Women’s Hospital, Herston, QLD 4029 Australia; 179 Hunter Area Pathology Service, John Hunter Hospital, Locked Bag 1 Regional Mail Centre, Randwick, NSW 2310 Australia; 180 Research Department, WEHI C/o Royal Melbourne Hospital, Parkville, VIC 3050 Australia; 181 Familial Cancer Centre, Royal Melbourne Hospital, Grattan Street, Parkville, VIC 3050 Australia; 182 Obstetrics and Gynecology, University of Auckland, Auckland, New Zealand; 183 The University of Queensland, Building 71/918, RBWH Campus, Herston, QLD 4029 Australia; 184 Cancer Unit, Queensland Institute of Medical Research, Herston, QLD 4029 Australia; 185 Familial Cancer and Genetics Medicine, Royal Melbourne Hospital, 2nd Floor Grattan Street, Parkville, VIC 3050 Australia; 186 Deputy Head, Cancer Program, Monash University Rm 349, Level 3, Building 76, 19 Innovation Walk, Clayton, VIC 3800 Australia; 187 Research Department, Peter MacCallum Cancer Centre, St Andrew’s Place, East Melbourne, VIC 3002 Australia; 188 University of NSW, Prince of Wales Hospital, Barker Street, Randwick, NSW 2031 Australia; 189 Heredity Cancer Clinic, Prince of Wales Hospital, Randwick, NSW 2031 Australia; 190 The Walter and Eliza Hall Institute of Medical Research, Post Office Royal Melbourne Hospital, Parkville, VIC 3050 Australia; 191 Molecular Cancer Epidemiology Laboratory, Queensland Institute of Medical Research, P.O. Royal Brisbane Hospital, Herston, QLD 4027 Australia; 192 Family Cancer Clinic, St Vincent’s Hospital, Darlinghurst, NSW 2010 Australia; 193 Department of Genetics, Royal Melbourne Hospital, Parkville, VIC 3050 Australia; 194 Genome.One, 370 Victoria St Darlinghurst, Darlinghurst, 2010 NSW Australia; 195 Staff Specialist, Prince of Wales Hereditary Cancer Centre, Level 1, Bright Building, Barker Street, Randwick, NSW 2031 Australia

**Keywords:** Cancer genetics, Breast cancer, Genetic association study

## Abstract

Evidence from literature, including the BRIDGES study, indicates that germline protein truncating variants (PTVs) in *FANCM* confer moderately increased risk of ER-negative and triple-negative breast cancer (TNBC), especially for women with a family history of the disease. Association between *FANCM* missense variants (MVs) and breast cancer risk has been postulated. In this study, we further used the BRIDGES study to test 689 *FANCM* MVs for association with breast cancer risk, overall and in ER-negative and TNBC subtypes, in 39,885 cases (7566 selected for family history) and 35,271 controls of European ancestry. Sixteen common MVs were tested individually; the remaining rare 673 MVs were tested by burden analyses considering their position and pathogenicity score. We also conducted a meta-analysis of our results and those from published studies. We did not find evidence for association for any of the 16 variants individually tested. The rare MVs were significantly associated with increased risk of ER-negative breast cancer by burden analysis comparing familial cases to controls (OR = 1.48; 95% CI 1.07–2.04; *P* = 0.017). Higher ORs were found for the subgroup of MVs located in functional domains or predicted to be pathogenic. The meta-analysis indicated that *FANCM* MVs overall are associated with breast cancer risk (OR = 1.22; 95% CI 1.08–1.38; *P* = 0.002). Our results support the definition from previous analyses of *FANCM* as a moderate-risk breast cancer gene and provide evidence that *FANCM* MVs could be low/moderate risk factors for ER-negative and TNBC subtypes. Further genetic and functional analyses are necessary to clarify better the increased risks due to *FANCM* MVs.

## Introduction

Since the discovery of the high-risk breast cancer predisposition genes *BRCA1* and *BRCA2*, extensive efforts have tried to identify additional breast cancer predisposition genes. Many candidate genes have been proposed but replication studies have been confirmatory for only few of them [1]. Recently, two large case-control studies were conducted in which several established and candidate breast cancer predisposition genes were tested. The BRIDGES study from the Breast Cancer Association Consortium (BCAC) tested 34 genes in 60,466 women with breast cancer and 53,461 controls [2]. In the second study, 28 genes were tested among 32,247 women with breast cancer and 32,544 unaffected women from US population-based studies in the CARRIERS consortium [3]. Results from both studies were concordant in confirming that germline protein truncating variants (PTVs) in *BRCA1*, *BRCA2* and *PALB2* are associated with high-risk of breast cancer, that PTVs in *CHEK2* and *ATM* confer moderate risk especially for the ER-positive disease subtype, and that PTVs in *RAD51C*, *RAD51D* and *BARD1* are moderate risk variants for ER-negative breast cancer. Lack of evidence of association was detected for PTVs in the great majority of the other tested candidate genes, but for one—namely *FANCM*—some evidence for association with ER-negative breast cancer was observed [2].

The association between a *FANCM* PTV and breast cancer risk was initially investigated in 2013 [4]. Since then, many case-control studies have been conducted, most based on the testing the three most common PTVs. Specifically, p.Gln1701* (c.5101 C > T) and p.Gly1906Alafs*12 (c.5791 C > T), which are expected to cause the loss of the FAAP24 binding domain in the FANCM protein C-terminus, were reported by a study of Finnish women as moderate risk variants for ER-negative and triple-negative breast cancer (TNBC) [5, 6]. In a large study of Caucasian women, we observed that the p.Arg658* (c.1972C > T), the third most common PTV, located in the protein N-terminus, was associated with moderate risk for ER-negative and TNBC subtypes, but the evidence of association for p.Gly1906Alafs*12 was inconclusive, and no evidence was observed for p.Gln1701* [7]. Overall, these and other studies [8, 9]—reviewed in Peterlongo et al. (2021)—indicate that *FANCM* PTVs are potential risk variants for ER-negative and TNBC subtypes; more precisely, they suggest that each PTV confers an increase risk with magnitude that may vary depending on its position in the gene or on the population genetic background [10].

While PTVs in breast cancer predisposition genes are usually considered bona fide pathogenic, missense variants (MVs) are often referred to as “variants of uncertain significance”. Their effect on protein function and cancer risk is generally unknown and difficult to estimate. Several in silico tools that predict pathogenicity of MVs have been developed that, together with additional evidence, such as frequency data, segregation analyses and functional assays, allow some MVs to be classified. However, MVs are often so infrequent that they have to be combined overall, or in subgroups based on their location in the gene domains or pathogenicity prediction score, in order to generate evidence of pathogenicity. MVs in several established and candidate breast cancer predisposition genes have been tested for association with breast cancer risk in many studies. To date, the potential association between *FANCM* MVs and breast cancer risk has been investigated by three studies in which all the rare variants were combined in burden analyses. Two studies were conducted using familial breast cancer cases with no *BRCA1* or *BRCA2* pathogenic variants and controls from the general population. The first, based on the analysis of 1207 cases and 1199 controls from France, did not find clear evidence of association with *FANCM* MVs (OR = 1.6; 95% CI 0.9–2.8) [11]. The second study, including 5770 cases and 5741 population-matched controls predominantly of European ancestry reported a statistically significant association with an OR of 1.50 (95% CI 1.16–1.93) [12]. In the third analysis, which was part of the BRIDGES study, rare *FANCM* MVs (allele frequency <0.1%) were tested in population- and family-based studies combined and separately. An association with breast cancer risk was found when comparing cases selected for family history of breast cancer and controls, with an OR estimate of 1.22 (95% CI 1.05–1.42) [2]. In the present study, we analysed further the BRIDGES data derived from the *FANCM* sequencing in women of European ancestry from population- and family-based studies. Specifically, we assessed 673 rare MVs with allele frequency <0.1% that were combined in burden analyses, but we also assessed individually 16 common MVs with allele frequency ≥0.1%. The burden analyses were based on the MVs’ gene domain location, and their pathogenicity prediction score. Analyses were conducted to assess associations with overall breast cancer but also the ER-negative and TNBC disease subtypes.

## Materials and methods

### Study sample

In this work we included women affected with breast cancer (cases) and unaffected women (controls) from 40 studies participating in the BRIDGES project (Supplementary Table S1), as previously described [2]. All 40 studies were approved by the relevant ethical review board and used appropriate consent procedures. Twenty-eight studies included cases unselected for breast cancer family history and are defined as “population-based studies”. The remaining 12 studies included cases selected because they had a family history of breast cancer, and are defined as “family-based studies”. All women included in this study were of European ancestry and older than 18 years at breast cancer diagnosis (cases) or interview (controls). We excluded women who, having a family history for breast cancer, were eligible for the *BRCA1* and *BRCA2* test and at the moment of the study enrollment were known to carry a pathogenic variant in these genes. We also excluded all carriers of *FANCM* PTVs and all women with one or more unknown *FANCM* MV genotypes. Thus, a total of 39,885 breast cancer cases (of which 91.6% were invasive cases, 6.2% in situ cases, and 2.2% cases of unknown invasiveness) and 35,271 controls were included in this study. Of the cases, 32,083 (80.4%) were from population-based studies and 7566 (19.0%) were from family-based studies; for the remaining 236 cases (0.6%) this information was not available. Of all cases, 5880 had ER-negative breast cancer and 2176 had TNBC.

### Sequencing, variant calling and classification

**Fig. 1:**
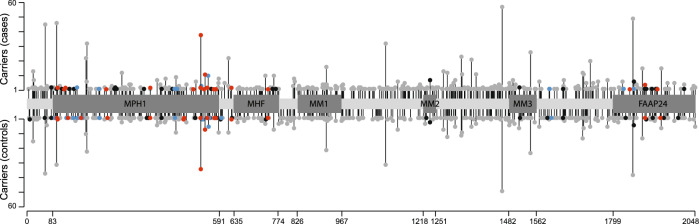
Representation of the 673 *FANCM* rare missense variants (MVs) with respect to the 2048 amino acid long FANCM protein. Functional and binding domains (MPH1, ATP-dependent DNA helicase; MHF, domain of interaction with the Histone Fold 1 and 2 (MHF1/2); MM1, motif of interaction with FANCF within the Fanconi Anemia core complex; MM2, motif of interaction with RecQ-Mediated genome Instability protein 1 (RMI1); MM3, highly conserved motif of still unknown function; FAAP24, domain of interaction with the Fanconi Anemia core complex-Associated Protein 24) are shown in dark grey and their boundaries indicated. The MVs are shown according to their position, the number of carriers in cases and controls, and by their in silico scores of pathogenicity according to BayesDel, CADD, Helix and REVEL tools; in grey are MVs predicted benign by all the tools; in black, MVs predicted pathogenic by one tool; in blue; MVs predicted pathogenic by two tools; in red, MVs predicted pathogenic by three or four tools.

The *FANCM* gene was included in a panel of 34 established and putative breast cancer predisposition genes that were sequenced in the context of the BRIDGES project [2]. Details of library preparation, next generation sequencing, variant calling, quality control procedures, and variant classification were described previously [2]. The *FANCM* MVs included in the present analyses were defined as common if their allelic frequency in controls was ≥0.1% and defined as rare if their allelic frequency in controls was <0.1%. The exact positions of *FANCM* functional and binding domains were derived from to UniProt database and published literature [13–15] (Fig. 1). Pathogenicity scores were assigned to each MV using the in silico prediction tools BayesDel [16], Combined Annotation Dependent Depletion (CADD) [17], Helix [18] and Rare Exome Variant Ensemble Learner (REVEL) [19]. The following cut-off were used to classify MVs as pathogenic: BayesDel score with MaxAF >0.069, CADD phred-scaled score ≥30, Helix score >0.50 and REVEL score >0.50.

### Statistical analyses

To test the association between *FANCM* MVs and breast cancer risk, we performed logistic regression analyses adjusting for country. Common MVs were tested individually by deriving allelic odds ratios (ORs) with their corresponding 95% confidence intervals (CIs) and *P values* (*P*). Multiple testing correction was applied using Benjamini and Hochberg procedure [20]. Rare MVs were tested by burden analyses deriving ORs (with 95% CIs) comparing variant carriers with non-carriers. In this case, heterozygous and homozygous carriers were not distinguished as the number of homozygous carriers was too small to be analysed separately. We first combined all rare variants together then grouped them based on their location within functional or binding domains and by pathogenicity score. Statistical analyses for both common and rare MVs were conducted using the full sample, and separately for population- or family-based studies, and for ER-negative and TNBC case subgroups (each compared to controls) separately. Finally, we performed a fixed-effect meta-analysis combining the OR that we derived in the analysis of family-based studies with the ORs derived by the two previously published studies conducted using familial cases [11, 12]. All statistical analyses were performed using STATA version 15.1 (StataCorp LLC, College Station, Texas, USA). All tests were two-sided and *P* < 0.05 were considered statistically significant.

## Results

A total of 689 unique *FANCM* MVs, of which 16 were common and 673 were rare, were detected in at least one woman from our study sample (Supplementary Table S2). All 16 common MVs were tested individually for association with breast cancer risk (Supplementary Table S3). Of these 16 MVs, seven showed a possible association (*P* < 0.05) with breast cancer risk or a protective effect in some of the case groups tested. But none were statistically significant after correction for multiple testing (Supplementary Table S3).

The 673 rare MVs are described and represented, based on their gene location, pathogenicity score according to four in silico tools, and the numbers of variant carriers in cases and controls, in Fig. 1. The burden analyses including all the rare 673 *FANCM* MVs did not indicate any statistically significant association with breast cancer risk either in the analysis of combined population- and family-based studies or when these groups were analysed separately (Table 1). The only significant association, with OR = 1.48 (95% CI 1.07–2.04; *P* = 0.017), was found with ER-negative breast cancer in the analysis of family-based studies. These analyses were repeated with subgroups of the variants. We firstly considered the subgroup of the 372 MVs located within the *FANCM* functional or binding domains but found no evidence of association. We then excluded the 76 MVs located in the FAAP24 domain and found that the 296 remaining MVs were associated with TNBC in familial studies with an OR = 2.27 (95% CI 1.15–4.47; *P* = 0.017). We further selected among the 296 MVs the 61 MVs predicted to be pathogenic by at least one of the four in silico tools used and found an association with TNBC with an OR = 3.51 (95% CI 1.07–11.44; *P* = 0.038) in the familial studies (Table 1).

**Table 1:** Association analyses of the 673 *FANCM* rare MVs with breast cancer risk overall and in ER-negative and TNBC subtypes tested in population- and family-based studies combined and separately.

	All studies		Population-based studies		Family-based studies	
	Carriers/Non-carriers (Freq%)	OR (95% CI); *P*	Carriers/Non-carriers (Freq%)	OR (95% CI); *P*	Carriers/Non-carriers (Freq%)	OR (95% CI); *P*
*All MVs [673]*						
Controls	1006/34,265 (2.85)	–	1006/34,265 (2.85)	–	1006/34,265 (2.85)	–
All cases	1193/38,692 (2.99)	1.04 (0.95–1.13); 0.396	932/31,151 (2.90)	1.02 (0.93–1.12); 0.637	254/7312 (3.36)	1.12 (0.97–1.30); 0.135
ER-negative	176/5704 (2.99)	1.02 (0.87–1.21); 0.784	133/4763 (2.72)	0.94 (0.78–1.13); 0.491	42/906 (4.43)	**1.48 (1.07**–**2.04); 0.017**
TNBC	57/2119 (2.62)	0.89 (0.68–1.17); 0.417	42/1813 (2.26)	0.78 (0.57–1.06); 0.112	15/302 (4.73)	1.58 (0.93–2.68); 0.088
*MVs within functional or binding domains [372]*						
Controls	572/34,699 (1.62)	–	572/34,699 (1.62)	–	572/34,699 (1.62)	–
All cases	654/39,231 (1.64)	1.00 (0.90–1.13); 0.921	515/31,568 (1.60)	0.99 (0.88–1.12); 0.877	134/7432 (1.77)	1.06 (0.87–1.30); 0.561
ER-negative	100/5780 (1.70)	1.04 (0.84–1.29); 0.724	77/4819 (1.57)	0.97 (0.76–1.23); 0.780	22/926 (2.32)	1.39 (0.90–2.16); 0.138
TNBC	29/2147 (1.33)	0.81 (0.56–1.19); 0.286	20/1835 (1.08)	0.66 (0.42–1.03); 0.070	9/308 (2.84)	1.72 (0.88–3.37); 0.115
*MVs within functional or binding domains excluding those in the FAAP24 BD [296]*						
Controls	440/34,831 (1.25)	–	440/34,831 (1.25)	–	440/34,831 (1.25)	–
All cases	509/39,376 (1.28)	1.01 (0.89–1.15); 0.815	398/31,685 (1.24)	0.99 (0.87–1.14); 0.952	107/7459 (1.41)	1.12 (0.89–1.40); 0.338
ER-negative	80/5800 (1.36)	1.08 (0.85–1.38); 0.528	63/4833 (1.29)	1.03 (0.79–1.34); 0.839	17/931 (1.79)	1.41 (0.86–2.31); 0.177
TNBC	26/2150 (1.19)	0.95 (0.64–1.42); 0.812	17/1838 (0.92)	0.73 (0.45–1.19); 0.209	9/308 (2.84)	**2.27 (1.15**–**4.47); 0.017**
*MVs within functional or binding domains excluding those in the FAAP24 BD and predicted pathogenic by at least one in silico tool [61]*						
Controls	92/35,179 (0.26)	–	92/35,179 (0.26)	–	92/35,179 (0.26)	–
All cases	107/39,778 (0.27)	1.04 (0.78–1.37); 0.803	81/32,002 (0.25)	0.96 (0.71–1.30); 0.809	25/7541 (0.33)	1.23 (0.76–1.99); 0.394
ER-negative	15/5865 (0.25)	0.98 (0.57–1.70); 0.950	10/4886 (0.20)	0.79 (0.41–1.52); 0.486	5/943 (0.53)	1.93 (0.76–4.89); 0.167
TNBC	4/2172 (0.18)	0.71 (0.26–1.94); 0.505	1/1854 (0.05)	0.21 (0.03–1.50); 0.120	3/314 (0.95)	**3.51 (1.07**–**11.44); 0.038**

The statistically significant results are indicated in bold. In square brackets is indicated the number of the MVs in each tested group.

*Freq* carrier frequency, *OR* odds ratio, *CI* confidence interval, *P* *p* value of association from *Z*-test, *TNBC* triple-negative breast cancer, *BD* binding domain.

**Fig. 2:**
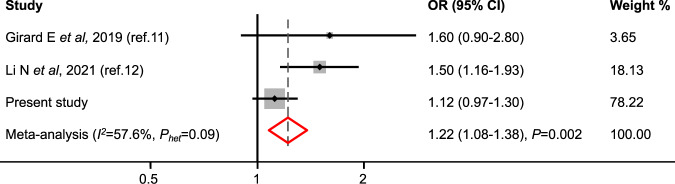
Meta-analysis of studies testing the association of *FANCM* MVs with familial breast cancer risk and based on the analysis of 14,543 familial breast cancer cases and 42,211 controls. OR odds ratio, CI confidence interval, *I*^*2*^ percentage of heterogeneity among the studies; *P*_*het*_, *p value* calculated using the Cochran’s *Q*-test for heterogeneity; *P, p value* of association from *Z*-test.

Finally, we considered the two studies published so far testing the association between *FANCM* MVs and breast cancer risk conducted using familial designs and excluding carriers of *BRCA1* or *BRCA2* pathogenic variants [11, 12]. Thus, we performed a meta-analysis combining results from these studies with those from our analysis and found that all *FANCM* MVs combined were associated with familial breast cancer risk with OR = 1.22 (95% CI 1.08–1.38; *P* = 0.002, Fig. 2).

## Discussion

In this study, we re-analysed the BRIDGES *FANCM* sequencing data assessing the breast cancer risk effects of 689 unique MVs in 39,885 European breast cancer cases and 35,271 controls from population- and family-based studies. According to their allele frequencies, these MVs were analysed either individually or by burden analyses, in the latter case combined in groups considering their gene domain location or their pathogenicity score. Also, the cases were analysed in different combinations, by study-design, and overall and for ER-negative or TNBC clinical subtypes.

Sixteen common MVs with an allele frequency ≥0.1% were analysed individually but we did not find evidence for association for any of these variants. The remaining 673 MVs were rare, with an allele frequency <0.1%. The best approach to study the risks conferred by these variants is that of combining single variant data in burden analysis and of conducting meta-analyses of different studies. Overall, our results and those from the previously conducted studies [2, 11, 12], indicate that *FANCM* MVs are associated with familial breast cancer risk, suggesting that these variants are low-risk susceptibility variants for breast cancer. This observation was confirmed by the meta-analysis of our and the published results [11, 12] showing that these variants were associated with familial breast cancer risk (OR = 1.22, Fig. 2). However, as studies with statistically significant results have increased likelihood of being published, we cannot exclude that this result is affected by the presence of publication bias. It is also interesting to note that a higher OR estimate, indicating moderately increased risk, was derived for the 296 MVs located within functional or binding domains excluding those in the FAAP24 (OR = 2.27, Table 1), and for the subgroup of 61 variants that among the 296 were predicted to be pathogenic by at least one of the in silico tools we used (OR = 3.51, Table 1). Further studies based on in vitro assays should be conducted to test if any of these MVs is functionally deleterious allowing to better clarify their risk effect on breast cancer. It should be noted that in the present study, as well as in the previously published ones, the association of *FANCM* MVs was only found in family-based studies. While this supports the hypothesis that *FANCM* MVs are breast cancer risk factors, the ORs we found in this study are an overestimate of the risks these variants confer. The lack of associations of *FANCM* MVs with breast cancer risk in the analyses of only population-based studies could be explained by the presence of other unmeasured risk variants aggregating in families that may interact with *FANCM* MVs. Results from the analysis of family- and population-based studies combined are similar to those of the analysis of only population-based studies as familial cases represent only the 19% of all the cases included.

In conclusion, our data suggest that at least some of the *FANCM* MVs—in particular those located in some gene domains and classified as pathogenic in silico—could be risk variants for ER-negative breast cancer in familial settings. Larger association studies and, functional assays may be helpful to better clarify these MVs effects on breast cancer risk. Overall, our results showed that perturbation of the *FANCM* gene has an impact on breast cancer risk, reinforcing the knowledge that *FANCM* is a breast cancer gene predisposing especially to develop ER-negative and TNBC disease subtypes.

## Supplementary information

Supplementary Table

Supplementary Table

## Data Availability

The datasets analysed in the current study are available via the BCAC Data Access Co-ordinating Committee (bcac@medschl.cam.ac.uk) upon reasonable request. Summary-level genotype data are available via http://bcac.ccge.medschl.cam.ac.uk and in Supplementary Table S2. Individual-level data are available via the BCAC Data Access Co-ordinating Committee (bcac@medschl.cam.ac.uk).
