# Supplementary material for: FANCM missense variants and breast cancer risk: a case-control association study of 75,156 European women
Source: Eur J Hum Genet. 2023 Jan 27;31(5):578–87. doi: 10.1038/s41431-022-01257-w (PMC10172381; doi:10.1038/s41431-022-01257-w)
Supplement: Supplementary file 1 — Supplementary Table [file 41431_2022_1257_MOESM1_ESM.pdf]

Supplementary Table S1. Description of the studies included in the present analysis.

Study	Abbreviation	Country	Study design	Familial cases included	No. of controls	No. of breast cancer cases	Total
Amsterdam Breast Cancer Study	ABCS	Netherlands	Hospital-based consecutive cases; population-based controls	No	1,611	987	2,598
Amsterdam Breast Cancer Study - Familial	ABCS-F	Netherlands	Clinical Genetic Center-based cases	Yes	0	194	194
Bavarian Breast Cancer Cases and Controls	BBCC	Germany	Hospital-based cases; population based controls	No	99	167	266
Breast Cancer in Galway Genetic Study	BIGGS	Ireland	Hospital-based cases; population based controls	No	357	357	714
Breast Oncology Galicia Network	BREOGAN	Spain	Population-based case-control	No	333	461	794
Breast Cancer Study of the University of Heidelberg	BSUCH	Germany	Hospital-based cases;healthy blood donator controls	No	375	228	603
Crete Cancer Genetics Program	CCGP	Greece	Hospital-based case-control study	No	262	401	663
CECILE Breast Cancer Study	CECILE	France	Population-based case-control study	No	810	737	1,547
Spanish National Cancer Centre Breast Cancer Study	CNIO-BCS	Spain	Case-control study	Yes	500	437	937
Family History Risk Study	FHRISK	UK	Clinic-based cohort study with a nested case-control study	Yes	172	72	244
German Consortium for Hereditary Breast & Ovarian Cancer	GC-HBOC	Germany	Clinic-based case study and prospective cohort study	Yes	1,519	2,238	3,757
Gene Environment Interaction and Breast Cancer in Germany	GENICA	Germany	Population-based case-control study	No	757	702	1,459
Generation Scotland	GENSCOT	Scotland	Prospective family-based cohort study; nested case-control	No	643	365	1,008
Genetic Epidemiology Study of Breast Cancer by Age 50	GESBC	Germany	Population-based study of women <50 years	No	912	455	1,367
Hannover Breast Cancer Study	HABCS	Germany	Hospital-based case-control study	No	802	943	1,745
Helsinki Breast Cancer Study	HEBCS	Finland	Hospital-based case-control study, plus additional familial cases	Yes	792	1,660	2,452
Hereditair Borst-en eierstokkanker Onderzoek Nederland	HEBON	Netherlands	Clinical genetic center-based recruitment of familial breast or ovarian cancer patients (cases)	Yes	0	1,358	1,358
Hannover- Minsk Breast Cancer Study	HMBCS	Belarus	Hospital-based cases; population based controls	No	156	278	434
Hannover-Ufa Breast Cancer Study	HUBCS	Russia	Hospital-based cases; population based controls	No	170	196	366
Karolinska Breast Cancer Study	KARBAC	Sweden	Population and hospital-based cases; geographically matched controls	Yes	420	344	764
Karolinska Mammography Project for Risk Prediction of	KARMA	Sweden	Cohort study	No	4,980	2,739	7,719

Breast Cancer - Cohort Study							
Kuopio Breast Cancer Project	KBCP	Finalnd	Population-based prospective clinical cohort	No	69	530	599
Kathleen Cuningham Foundation Consortium for research into Familial Breast Cancer/Australian Ovarian Cancer Study	KCONFAB/AOCS	Australia and New Zealand	Clinic-based recruitment of familial breast cancer patients (cases); population-based case-control study of ovarian cancer (controls only)	Yes	5	1,019	1,024
Mammary Carcinoma Risk Factor Investigation	MARIE	Germany	Population-based case-control study	No	1,514	1,969	3,483
Cyprus Breast Cancer Case Control Study	MASTOS	Cyprus	Population-based case-control study	No	1,041	937	1,978
Milan Breast Cancer Study Group	MBCSG	Italy	Clinic-based recruitment of familial/early onset breast cancer patients (cases); population-based controls	Yes	685	865	1,550
Melbourne Collaborative Cohort Study	MCCS	Australia	Prospective cohort study: nested case-control study	No	976	977	1,953
Norwegian Breast Cancer Study	NBCS	Norway	Hospital-based case-control study	No	584	548	1,132
Ontario Familial Breast Cancer Registry	OFBCR	Canada	Population-based familial case-control study	Yes	369	447	816
Leiden University Medical Centre Breast Cancer Study	ORIGO	Netherlands	Hospital-based prospective cohort study	No	877	0	877
NCI Polish Breast Cancer Study	PBCS	Poland	Population-based case-control study	No	1,731	1,642	3,373
Karolinska Mammography Project for Risk Prediction of Breast Cancer - Case-Control Study	PKARMA	Sweden	Population-based study	No	0	23	23
The Prostate,Lung,Colorectal and Ovarian (PLCO) Cancer Screening Trial	PLCO	USA	Prospective cohort study: nested case-control	No	1,903	1,803	3,706
Predicting the Risk Of Cancer At Screening Study	PROCAS	UK	Population based study	No	1,151	403	1,554
Rotterdam Breast Cancer Study	RBCS	Netherlands	Hospital-based case-control study, Rotterdam area	Yes	883	1,017	1,900
Singapore and Sweden Breast Cancer Study	SASBAC	Sweden	Population-based case-control study	No	1,240	1,046	2,286
Study of Epidemiology and Risk factors in Cancer Heredity	SEARCH	UK	Population-based case-control study	No	6,108	9,447	15,555
Städtisches Klinikum Karlsruhe Deutsches Krebsforschungs zentrum Study	SKKDKFZS	Germany	Hospital-based breast cancer cohort	No	0	845	845
IHCC-Szczecin Breast Cancer Study	SZBCS	Poland	Hospital-based case-control study	No	187	340	527
Utah Breast Cancer Study	UBCS	USA	Population-based and hospital-based case-control study, plus additional familial cases	Yes	278	708	986
All studies					35,271	39,885	75,156

Supplementary Table S3. Association analyses of the 16 *FANCM* common MVs with breast cancer risk overall and in ER-negative and TNBC subtypes tested in population- and family-based studies combined and separately.

		**All studies**		**Population-based studies**		**Family-based studies**	
**Common MVs**	P_{HWE}	**No. of rare/common alleles (MAF)**	**OR (95% CI); *P; P***	**No. of rare/common alleles (MAF)**	**OR (95% CI); *P; P***	**No. of rare/common alleles (MAF)**	**OR (95% CI); *P; P***
**c.171G>C, p.Leu57Phe**							
Controls	0.86	199/70,343 (0.28)	-	199/70,343 (0.28)	-	199/70,343 (0.28)	-
All cases		231/79,539 (0.29)	1.04 (0.86-1.26); 0.695; 0.949	175/63,991 (0.27)	0.96 (0.78-1.18); 0.718; 0.949	56/15,076 (0.37)	**1.47 (1.07-2.02); 0.017; 0.524**
ER-negative		38/11,722 (0.32)	1.19 (0.84-1.68); 0.338; 0.801	28/9,764 (0.29)	1.04 (0.69-1.54); 0.861; 0.954	10/1,886 (0.53)	**2.14 (1.11-4.10); 0.022; 0.524**
TNBC		17/4,335 (0.39)	1.44 (0.88-2.38); 0.148; 0.678	11/3,699 (0.30)	1.08 (0.59-1.99); 0.795; 0.950	6/628 (0.95)	**3.88 (1.69-8.87); 0.001; 0.071**
**c.229A>G, p.Thr77Ala**							
Controls	0.22	924/69,618 (1.31)	-	924/69,618 (1.31)	-	924/69,618 (1.31)	-
All cases		1,000/78,770 (1.25)	0.97 (0.89-1.06); 0.554; 0.921	820/63,346 (1.28)	0.97 (0.88-1.07); 0.546; 0.921	175/14,957 (1.16)	0.93 (0.79-1.11); 0.440; 0.878
ER-negative		131/11,629 (1.11)	0.87 (0.72-1.04); 0.134; 0.678	99/9,693 (1.01)	**0.78 (0.63-0.96); 0.021; 0.524**	32/1,864 (1.69)	1.35 (0.94-1.93); 0.107; 0.665
TNBC		49/4,303 (1.13)	0.88 (0.66-1.18); 0.397; 0.847	44/3,666 (1.19)	0.92 (0.68-1.25); 0.609; 0.938	5/629 (0.79)	0.62 (0.26-1.51), 0.296; 0.783
**c.524C>T, p.Ser175Phe**							
Controls	0.41	4,204/66,338 (5.96)	-	4,204/66,338 (5.96)	-	4,204/66,338 (5.96)	-
All cases		4,710/75,060 (5.90)	0.98 (0.94-1.02); 0.348; 0.801	3,836/60,330 (5.98)	1.01 (0.96-1.05); 0.778; 0.949	845/14,287 (5.58)	**0.87 (0.80-0.95); 0.001; 0.071**
ER-negative		709/11,051 (6.03)	0.98 (0.91-1.07); 0.708; 0.949	588/9,204 (6.00)	0.99 (0.91-1.08); 0.835; 0.954	117/1,779 (6.17)	0.97 (0.91-1.17); 0.630; 0.938
TNBC		275/4,077 (6.32)	1.03 (0.91-1.17); 0.630; 0.938	235/3,475 (6.33)	1.04 (0.91-1.19); 0.541; 0.921	39/595 (6.15)	0.96 (0.69-1.33); 0.811; 0.951
**c.527C>T, p.Thr176Ile**							
Controls	0.90	353/70,189 (0.50)	-	353/70,189 (0.50)	-	353/70,189 (0.50)	-
All cases		425/79,345 (0.53)	1.05 (0.91-1.21); 0.481; 0.920	351/63,815 (0.55)	1.10 (0.95-1.27); 0.218; 0.678	72/15,060 (0.48)	0.86 (0.65-1.12); 0.266; 0.757
ER-negative		76/11,684 (0.65)	1.24 (0.97-1.60); 0.086; 0.653	61/9,731 (0.62)	1.22 (0.93-1.60); 0.156; 0.678	14/1,882 (0.74)	1.32 (0.76-2.28); 0.318; 0.801
TNBC		31/4,321 (0.71)	1.37 (0.94-1.98); 0.096; 0.653	25/3,685 (0.67)	1.31 (0.87-1.97); 0.195; 0.678	6/628 (0.95)	1.68 (0.74-3.80); 0.214; 0.678
**c.624A>G, p.Ile208Met**							
Controls	0.58	1,100/69,442 (1.56)	-	1,100/69,442 (1.56)	-	1,100/69,442 (1.56)	-
All cases		1,158/78,621 (1.45)	0.95 (0.87-1.03); 0.204; 0.678	913/63,253 (1.42)	**0.91 (0.83-0.99); 0.029; 0.584**	238/14,894 (1.57)	1.14 (0.98-1.33); 0.076; 0.654
ER-negative		167/11,593 (1.42)	0.95 (0.81-1.13); 0.590; 0.938	143/9,649 (1.46)	0.96 (0.81-1.15); 0.685; 0.949	23/1,873 (1.21)	0.87 (0.57-1.33); 0.531; 0.921
TNBC		59/4,293 (1.36)	0.91 (0.70-1.19); 0.512; 0.921	53/3,647 (1.43)	0.95 (0.72-1.25); 0.721; 0.949	6/628 (0.95)	0.68 (0.30-1.53); 0.353; 0.801
**c.1237T>C, p.Tyr413His**							
Controls	0.76	113/70,429 (0.16)	-	113/70,429 (0.16)	-	113/70,429 (0.16)	-
All cases		126/79,644 (0.16)	1.02 (0.79-1.31); 0.899; 0.965	95/64,071 (0.15)	0.91 (0.70-1.20); 0.528; 0.921	31/15,101 (0.20)	**1.65 (1.08-2.53); 0.020; 0.524**
ER-negative		19/11,741 (0.16)	1.09 (0.67-1.78); 0.724; 0.949	14/9,778 (0.14)	0.94 (0.54-1.64); 0.824; 0.954	5/1,891 (0.26)	2.18 (0.87-5.47); 0.096; 0.654
TNBC		6/4,346 (0.14)	0.95 (0.42-2.17); 0.904; 0.965	4/3,706 (0.11)	0.72 (0.27-1.97); 0.528; 0.921	2/632 (0.32)	2.61 (0.63-10.78); 0.185; 0.678
**c.1576C>G, p.Leu526Val**							
Controls	0.80	94/70,448 (0.13)	-	94/70,448 (0.13)	-	94/70,448 (0.13)	-
All cases		124/79,646 (0.16)	1.20 (0.92-1.57); 0.176; 0.678	99/64,067 (0.15)	1.15 (0.86-1.52); 0.339; 0.801	25/15,107 (0.17)	1.26 (0.78-2.03); 0.342; 0.801
ER-negative		18/11,742 (0.15)	1.18 (0.71-1.96); 0.527; 0.921	16/9,776 (0.16)	1.25 (0.73-2.12); 0.411; 0.864	2/1,894 (0.11)	0.75 (0.18-3.09); 0.686; 0.949
TNBC		4/4,348 (0.09)	0.70 (0.26-1.91); 0.489; 0.920	4/3,706 (0.11)	0.82 (0.30-2.23); 0.698; 0.949	0/634 (0.00)	Na
**c.1964A>G, p.Asn655Ser**							
Controls	0.19	1,135/69,407 (1.61)	-	1,135/69,407 (1.61)	-	1,135/69,407 (1.61)	-
All cases		1,238/78,532 (1.55)	0.98 (0.90-1.06); 0.627; 0.938	1,000/63,166 (1.56)	0.96 (0.88-1.05); 0.393; 0.847	230/14,902 (1.52)	1.01 (0.87-1.18); 0.856; 0.954
ER-negative		182/11,578 (1.55)	1.00 (0.85-1.17); 0.980; 0.988	149/9,643 (1.52)	0.97 (0.82-1.15); 0.742; 0.949	32/1,864 (1.69)	1.15 (0.80-1.65); 0.434; 0.878
TNBC		70/4,282 (1.61)	1.04 (0.82-1.33); 0.728; 0.949	57/3,653 (1.54)	0.98 (0.75-1.29); 0.926; 0.973	13/621 (2.05)	1.41 (0.81-2.47); 0.222; 0.678
**c.2632G>T, p.Val878Leu**							
Controls	0.03	8,154/62,388 (11.56)	-	8,154/62,388 (11.56)	-	8,154/62,388 (11.56)	-
All cases		9,254/70,516 (11.60)	1.00 (0.97-1.03); 0.946; 0.987	7,444/56,722 (11.60)	1.00 (0.97-1.04); 0.780; 0.949	1,740/13,392 (11.50)	0.96 (0.91-1.02); 0.190; 0.678
ER-negative		1,380/10,380 (11.73)	1.01 (0.95-1.07); 0.809; 0.950	1,144/8,648 (11.68)	1.01 (0.94-1.07); 0.842; 0.954	225/1,671 (11.87)	1.00 (0.87-1.16); 0.969; 0.989
TNBC		530/3,822 (12.18)	1.05 (0.95-1.15); 0.320; 0.801	453/3,257 (12.21)	1.05 (0.95-1.17); 0.293; 0.784	75/559 (11.83)	1.00 (0.78-1.27); 0.995; 0.995

**c.2859A>C, p.Lys953Asn**							
Controls	0.66	166/70,376 (0.24)	-	166/70,376 (0.24)	-	166/70,376 (0.24)	-
All cases		158/79,612 (0.20)	0.85 (0.68-1.06); 0.154; 0.678	133/64,033 (0.21)	0.88 (0.70-1.10); 0.265; 0.757	25/15,107 (0.17)	0.74 (0.48-1.15); 0.183; 0.678
ER-negative		29/11,731 (0.25)	1.06 (0.71-1.58); 0.767; 0.949	22/9,770 (0.22)	0.96 (0.61-1.50); 0.861; 0.954	7/1,889 (0.37)	1.65 (0.76-3.58); 0.201; 0.678
TNBC		12/4,340 (0.28)	1.19 (0.66-2.15); 0.553; 0.921	10/3,700 (0.27)	1.16 (0.61-2.20); 0.647; 0.947	2/632 (0.32)	1.38 (0.34-5.65); 0.649; 0.947
**c.3758A>G, p.Asn1253Ser**							
Controls	0.04	2,391/68,151 (3.39)	-	2,391/68,151 (3.39)	-	2,391/68,151 (3.39)	-
All cases		2,726/77,044 (3.42)	1.00 (0.94-1.06); 0.978; 0.988	2,204/61,962 (3.43)	1.02 (0.96-1.08); 0.579; 0.938	512/14,620 (3.38)	0.99 (0.90-1.10); 0.922; 0.947
ER-negative		437/11,323 (3.72)	1.10 (0.99-1.22); 0.082; 0.654	364/9,428 (3.72)	1.10 (0.98-1.23); 0.104; 0.665	71/1,825 (3.74)	1.10 (0.86-1.40); 0.442; 0.878
TNBC		165/4,187 (3.79)	1.12 (0.95-1.31); 0.174; 0.678	144/3,566 (3.88)	1.14 (0.96-1.36); 0.120; 0.678	21/613 (3.31)	0.97 (0.63-1.50); 0.895; 0.965
**c.4378A>G, p.Ile1460Val**							
Controls	0.05	7,255/63,287 (10.28)	-	7,255/63,287 (10.28)	-	7,255/63,287 (10.28)	-
All cases		8,294/71,476 (10.40)	1.01 (0.97-1.04); 0.689; 0.949	6,659/57,507 (10.38)	1.01 (0.98-1.05); 0.530; 0.921	1,571/13,561 (10.38)	0.97 (0.91-1.03); 0.287; 0.784
ER-negative		1,255/10,505 (10.67)	1.03 (0.96-1.09); 0.394; 0.847	1,050/8,742 (10.72)	1.04 (0.97-1.11); 0.270; 0.757	194/1,702 (10.23)	0.96 (0.83-1.12); 0.625; 0.938
TNBC		483/3,869 (1.10)	1.07 (0.97-1.18); 0.166; 0.678	411/3,299 (11.08)	1.07 (0.97-1.19); 0.182; 0.678	70/564 (11.04)	1.05 (0.82-1.35); 0.708; 0.959
**c.4799C>T, p.Thr1600Ile**							
Controls	0.74	1,818/68,724 (2.58)	-	1,818/68,724 (2.58)	-	1,818/68,724 (2.58)	-
All cases		2,190/77,580 (2.75)	**1.07 (1.00-1.14); 0.034; 0.584**	1,760/62,406 (2.74)	1.06 (0.99-1.14); 0.064; 0.654	419/14,713 (2.77)	**1.12 (1.00-1.26); 0.049; 0.584**
ER-negative		335/11,425 (2.85)	**1.13 (1.00-1.27); 0.046; 0.584**	284/9,508 (2.90)	**1.14 (1.00-1.29); 0.043; 0.584**	49/1,847 (2.58)	1.07 (0.80-1.44); 0.624; 0.938
TNBC		123/4,229 (2.83)	1.12 (0.93-1.35); 0.215; 0.678	110/3,600 (2.96)	1.17 (0.96-1.42); 0.112; 0.667	13/621 (2.05)	0.85 (0.49-1.48); 0.575; 0.938
**c.5224A>G, p.Ile1742Val**							
Controls	0.003	863/69,679 (1.22)	-	863/69,679 (1.22)	-	863/69,679 (1.22)	-
All cases		981/78,789 (1.23)	0.99 (0.90-1.08); 0.797; 0.950	780/63,386 (1.22)	1.00 (0.90-1.10); 0.973; 0.988	195/14,937 (1.29)	0.97 (0.82-1.15); 0.745; 0.949
ER-negative		160/11,600 (1.36)	1.07 (0.90-1.27); 0.436; 0.878	131/9,661 (1.34)	1.07 (0.89-1.29); 0.482; 0.920	27/1,869 (1.42)	1.06 (0.72-1.56); 0.774; 0.949
TNBC		66/4,286 (1.52)	1.19 (0.92-1.53); 0.181; 0.678	57/3,653 (1.54)	1.22 (0.93-1.60); 0.150; 0.678	9/625 (1.42)	1.04 (0.54-2.03); 0.893; 0.965
**c.5434C>G, p.Pro1812Ala**							
Controls	0.07	7,407/63,135 (10.50)	-	7,407/63,135 (10.50)	-	7,407/63,135 (10.50)	-
All cases		8,453/71,317 (10.60)	1.00 (0.97-1.04); 0.783; 0.949	6,784/57,382 (10.57)	1.01 (0.97-1.04); 0.616; 0.938	1,604/13,528 (10.60)	0.96 (0.91-1.03); 0.260; 0.757
ER-negative		1,281/10,479 (10.89)	1.03 (0.96-1.09); 0.394; 0.847	1,069/8,723 (10.92)	1.04 (0.97-1.11); 0.304; 0.790	201/1,695 (10.60)	0.98 (0.84-1.13); 0.767; 0.949
TNBC		490/3,862 (11.26)	1.06 (0.96-1.17); 0.208; 0.678	417/3,293 (11.24)	1.07 (0.96-1.18); 0.223; 0.678	71/563 (11.20)	1.04 (0.81-1.34); 0.750; 0.949
**c.5627A>G, p.Asn1876Ser**							
Controls	0.04	2,361/68,181 (3.35)	-	2,361/68,181 (3.35)	-	2,361/68,181 (3.35)	-
All cases		2,701/77,069 (3.39)	1.00 (0.95-1.06); 0.898; 0.965	2,186/61,980 (3.41)	1.02 (0.96-1.08); 0.485; 0.920	504/14,628 (3.33)	1.00 (0.90-1.11); 0.981; 0.988
ER-negative		437/11,323 (3.72)	**1.11 (1.00-1.24); 0.043; 0.584**	364/9,428 (3.72)	1.11 (0.99-1.25); 0.061; 0.654	71/1,825 (3.74)	1.12 (0.88-1.43); 0.351; 0.801
TNBC		167/4,185 (3.84)	1.15 (0.8-1.35); 0.087; 0.654	145/3,565 (3.91)	1.17 (0.99-1.39); 0.070; 0.654	22/612 (3.47)	1.04 (0.68-1.60); 0.853; 0.954

Statistically significant results at *P*<0.05 are indicated in bold

P_{HWE}, *p-value* of Hardy-Weinberg Equilibrium; No., number; MAF, minor alllele frequency; OR, odds ratio; CI, confidence interval; *P*, *p-value* of association from *Z*-test; P_{corr}, *p-value* corrected using the Benjamini and Hochberg procedure; TNBC, triple-negative breast cancer; na, not applicable.
